# OSC-CO^2^: coattention and cosegmentation framework for plant state change with multiple features

**DOI:** 10.3389/fpls.2023.1211409

**Published:** 2023-10-31

**Authors:** Rubi Quiñones, Ashok Samal, Sruti Das Choudhury, Francisco Muñoz-Arriola

**Affiliations:** ^1^ School of Computing, University of Nebraska-Lincoln, Lincoln, NE, United States; ^2^ Computer Science Department, Southern Illinois University Edwardsville, Edwardsville, IL, United States; ^3^ School of Natural Resources, University of Nebraska-Lincoln, Lincoln, NE, United States; ^4^ Department of Biological Systems Engineering, University of Nebraska-Lincoln, Lincoln, NE, United States

**Keywords:** segmentation, cosegmentation, image analysis, high-throughput plant phenotyping, image sequences, object state change, multiple features, multiple dimensions

## Abstract

Cosegmentation and coattention are extensions of traditional segmentation methods aimed at detecting a common object (or objects) in a group of images. Current cosegmentation and coattention methods are ineffective for objects, such as plants, that change their morphological state while being captured in different modalities and views. The Object State Change using Coattention-Cosegmentation (OSC-CO^2^) is an end-to-end unsupervised deep-learning framework that enhances traditional segmentation techniques, processing, analyzing, selecting, and combining suitable segmentation results that may contain most of our target object’s pixels, and then displaying a final segmented image. The framework leverages coattention-based convolutional neural networks (CNNs) and cosegmentation-based dense Conditional Random Fields (CRFs) to address segmentation accuracy in high-dimensional plant imagery with evolving plant objects. The efficacy of OSC-CO^2^ is demonstrated using plant growth sequences imaged with infrared, visible, and fluorescence cameras in multiple views using a remote sensing, high-throughput phenotyping platform, and is evaluated using Jaccard index and precision measures. We also introduce CosegPP+, a dataset that is structured and can provide quantitative information on the efficacy of our framework. Results show that OSC-CO^2^ out performed state-of-the art segmentation and cosegmentation methods by improving segementation accuracy by 3% to 45%.

## 1 Introduction

Segmentation is a widely used technique to extract the foreground object from the background before information extraction (Langan et al., 1998; Rezaee et al., 2000; Patz and Preusser, 2012; Fan et al., 2018; Liu et al., 2021). Image segmentation has been used in many application domains, including medicine (Das and Kundu, 2013; Lian et al., 2019; Zhou et al., 2019), traffic safety (Alessandretti et al., 2007; Chang et al., 2019; Chen et al., 2020), and earth system diagnostics (Hoerser and Kuenzer, 2020). However, the success of segmentation algorithms has been limited by the complexity and diversity of the imagery. Cosegmentation is a technique developed to address the problem of segmenting an object in a set of images (Rother et al., 2006; Tao et al., 2015; Ren et al., 2018; Tao et al., 2019). Since its introduction, it has been used in many domains, including plant imagery (Quiñones et al., 2021), PET-CT images (Zhong et al., 2019), and video-based person re-identification (Subramaniam et al., 2019).

Current cosegmentation methods have been developed for RGB images (Chen et al., 2014a; Lin et al., 2014; Subramaniam et al., 2019) for objects with no defined quantitative or qualitative features (e.g., environmental conditions, perspectives, temporality, among others.) (Quiñones et al., 2021). Currently, datasets lack specific labeling for cosegmentation, limiting the success and application of these visualization methods. Furthermore, some methods are dependent on training data which make them tedious to generate and time consuming for training (Chen et al., 2014b; Meng et al., 2016; Hsu et al., 2019).

Although engineered features, such as Scale-Invariant Feature Transform (Lowe, 2004) and Histogram of Oriented Gradients (Dalal and Triggs, 2005), have been widely used in conventional cosegmentation methods, they are no longer optimal cosegmentation analytics due to their pre-designed network features. Convolutional neural networks (CNNs), on the other hand, have demonstrated their effectiveness in producing feature extraction in image pairs (Krizhevsky et al., 2012). Yuan et al (Yuan et al., 2017). proposed a CNN-based supervised method for object cosegmentation that would produce the masks for an object in a pair of images. However, their method requires additional training data for the CNN model in the form of object masking.

With the explosion in variety, velocity, and volume of plant imagery datasets, traditional segmentation algorithms grapple with processing images and achieving high accuracy due to challenges, including occlusion and overlap. Cosegmentation algorithms have the potential to overcome these issues, but they only achieve high accuracy for the dataset with which they were trained.

**Figure 1:**
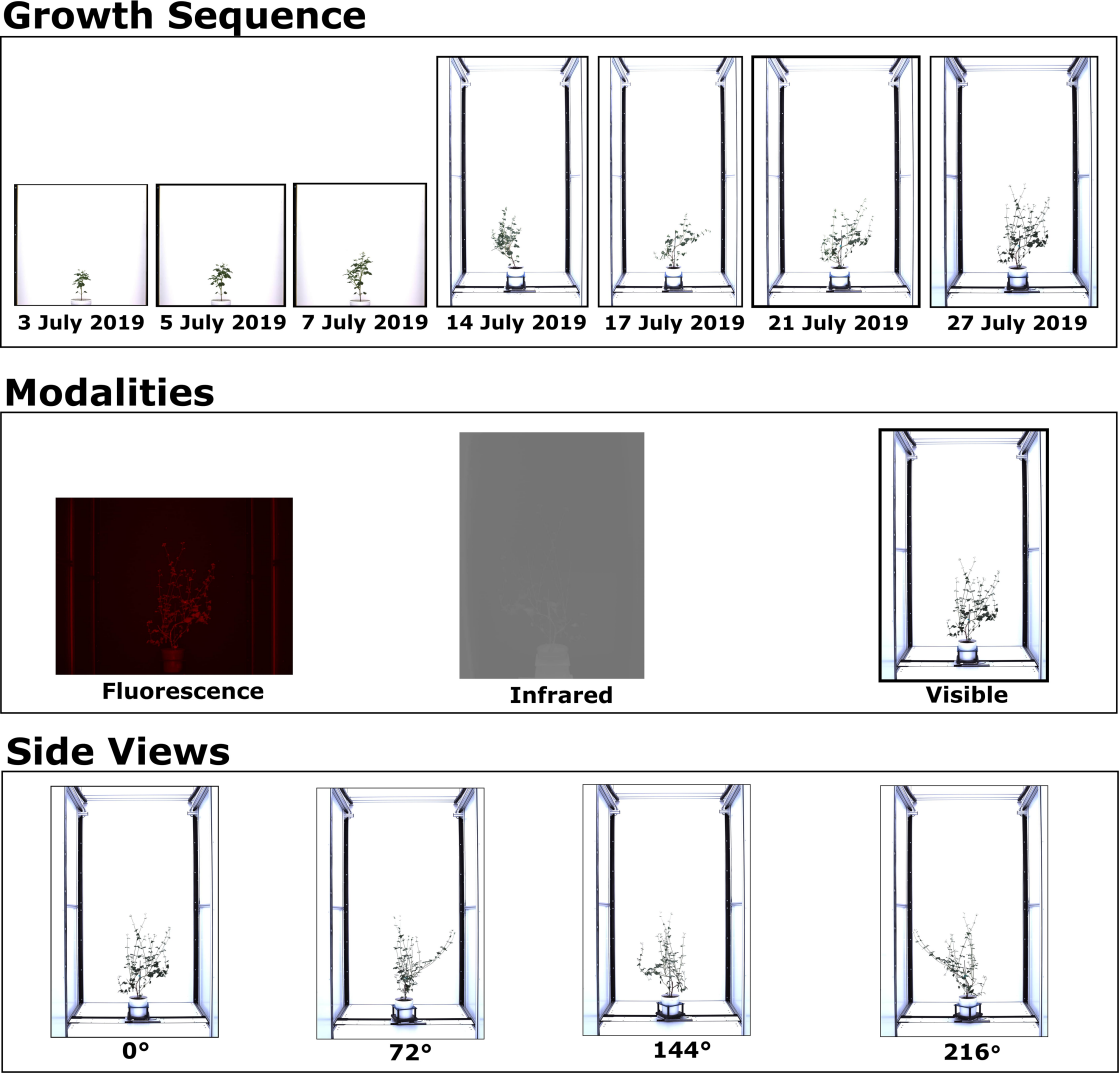
A preview of a VSTEM Dataset. This work will use the CosegPP dataset (Quiñones et al., 2021) and modify it as CosegPP+ and categorize it as a VSTEM dataset for our problem definition. The first row shows the growth sequence of a Buckwheat plant from 3^rd^ July 2019 to 27^th^ July 2019. The second row shows the three modality types of a Buckwheat plant on 27^th^ July 2019. The third row shows the four different side views of a Buckwheat plant on 27^th^ July 2019.

This paper presents Object State Change using Coattention-Cosegmentation (OSC-CO^2^), an end-to-end trainable unsupervised coattention- and cosegmentation-based framework for the increased segmentation of multiple feature objects that undergo state changes in high-throughput datasets. The state is referred to the object’s (plant’s) shape, orientation, and size at a specific point in time. OSC-CO^2^ is designed to process datasets that contain a variety of features, such as perspective (V), species (S), temporality (T), environmental conditions (E) and modality (M) (VSTEM) (**Figure 1**). The code for OSC-CO^2^ is publicly available at: https://github.com/rubiquinones/OSC-CO2.

OSC-CO^2^ is systematically evaluated using a VSTEM dataset retrieved from a high throughput plant phenotyping facility at the University of Nebraska-Lincoln, USA. This paper will use CosegPP+ [an extension of CosegPP (Quiñones et al., 2021)], which consists of growth sequences of multiple plants in their early growth period during which the plant grows, changes shape and appearance, develops new organs (leaves), and sometimes even rotates about a vertical axis for optimal light interception. The plants are grown in a greenhouse and are imaged daily in a high-throughput imaging platform using infrared, fluorescence, and RGB cameras with multiple views. Thus, the dataset exemplifies the VSTEM imagery and is manifestly more challenging than any dataset used in cosegmentation research because it categorizes the many features that pertain to the object while other datasets are random in their object selection with undefined features. The CosegPP+ dataset is available at: https://doi.org/10.5281/zenodo.6863013.

The specific contributions of this research include:

• An end-to-end unsupervised deep learning algorithm, OSC-CO^2^, to cosegment VSTEM imagery,
• Novel cosegmentation and temporal loss functions to adapt to the challenges of a high volume and variety VSTEM dataset,
• The demonstration of the efficacy of OSC-CO^2^ in a complex application domain – plant phenotyping. What makes plant phenotyping a complex domain is the dynamic growth and environmental interaction of an object (plant).

The literature is summarized in the next section, with a discussion on open problems, dataset biases, and how OSC-CO^2^ addresses these research gaps. The metrics used to evaluate the efficacy of the cosegmentation algorithms are also briefly described. Section 3 presents the problem definition, introduces our overall framework, and provides details of the different parts of the framework. Section 4 discusses the quantitative and qualitative results based on our evaluation using the precision and Jaccard index measures. Section 5 provides a final discussion, our conclusions, and potential directions for future work.

## 2 Literature review

This section summarizes the traditional and learning-based approaches for segmentation and cosegmentation algorithms and the datasets used for their evaluation. We also identify the research gaps and how our work addresses some of them.

### 2.1 Traditional segmentation algorithms

Traditional segmentation algorithms use a sequence of common image-processing steps to obtain a semantic and instant region of interest in an image. One technique is called frame differencing and is most used in high-throughput plant phenotyping systems since it is capable of imaging plants in a constant fixed camera, and lighting. It has been used by researchers (Woebbecke et al., 1992; Das Choudhury et al., 2017; Choudhury et al., 2018; Das Choudhury et al., 2020) by subtracting a fixed background image that only includes an empty pot, from an image that includes the plant while ensuring the background is constant. Although the segmentation technique is quick and low in computational demands, it does require images to come from a high-throughput system and is susceptible to residual noise around the plant region.

Color-based segmentation can address the issue of residual noise in imagery while having the flexibility to be used without a high-throughput system. This technique partitions parts of the image into different color regions based on its color features while assuming the color features are homogenous. Different color-based segmentations algorithms use either RGB (red, green, and blue), Lab (where L represents lightness, a indicates the red (positive) or green (negative), and b represents the yellow (positive) and blue (negative), or HSV (hue, saturation, and value) depending on the color space application. It was first inspired by Woebbecke et al. (1992), which used the green and red channels to derive the normalized difference index. Then, other researchers leveraged this idea by utilizing the red channel to derive the excess red index (Meyer and Neto, 2008), red, green, and blue channels to derive the color index of vegetation (Kataoka et al., 2003), and others (Hunt et al., 2005; Meyer and Neto, 2008; Zheng et al., 2009). It is a common technique to use for semantic analysis since a majority of a plant can be green compared to the background, but it does not handle multiple-colored objects (common in stress-induced plants) or when the background color is like the object.

Shape modeling-based segmentation is commonly used for leaf or stem semantic analysis (Yin et al., 2014; Agapito et al., 2015; Thorp et al., 2016; Chen et al., 2017; Li et al., 2017; Chen et al., 2019; Roggiolani et al., 2023; Williams et al., 2023), flower instant analysis (Thorp et al., 2016; Zhang et al., 2022; Mahajan et al., 2023), and fruits (Grift et al., 2017; Fu et al., 2019). Chen et al. (2017) had RGB images as an input to their framework, where it transformed the images into a polar coordinate system by using a plant’s density center as the origin. Thorp et al. (2016) used RGB images as input and transformed them into an HSI (hue, saturation, intensity) color space and then segmented the flowers using a Monte Carlo approach. Pape and Klukas (2015) attempted to reduce the impacts of illumination variability by modeling 3D histograms of LAB color space to aid in the segmentation process for rosette plants. Scharr et al. (2016) applied a super pixel-based unsupervised approach that can extract various regions of interests by implementing a seeded region growing algorithm. These techniques usually do not produce a high segmentation accuracy with varying accuracies of 40% to 80% (with superficial image edits, such as cropping, to improve the segmentation).

In addition to color being an obstacle for segmenting the region of interest from the background, the irregular physical characteristics of a plant and inconsistencies in lighting during data acquisition limit the effectiveness of simple, traditional segmentation methods. Therefore, approaches based on thresholding (Otsu, 1979; Sezgin and Sankur, 2004; Meng et al., 2019), frame differencing (Choudhury et al., 2018), color-based (Woebbecke et al., 1992; Zheng et al., 2009), and morphological operations (Zhou et al., 2021) have, in general, proven to be ineffective for segmentation in high-throughput plant imaging datasets. These techniques are unable to overcome image acquisition inconsistencies, including lighting variation, shadows, and plant positions, and more significantly, do not consider or leverage the dynamic nature of the plants’ evolving physical characteristics (Choudhury et al., 2018; Choudhury, 2020).

### 2.2 Learning-based segmentation algorithms

Learning-based segmentation algorithms are the preferred method especially since traditional segmentation algorithms often yield unsatisfactory results due to the plant being a complex object (Yin et al., 2014). Traditional segmentation algorithms also have issues overcoming common computer vision problems such as occlusion and large-scale lighting variations.

Clustering-based segmentation is a classification technique that attempts to find relational information among pixels in an image and classify them based on a similarity measure. These algorithms are the prerequisite for pursuing further complex phenotypic traits. They can eliminate noisy spots (Lee et al., 2018; Guo et al., 2021) and obtain homogenous regions (Ojeda-Magaña et al., 2010; Liu et al., 2018). Some segmentation algorithms target the semantic segmentation of plants, while others target the instant segmentation of plant parts, such as leaves, flowers, and fruit. Liu et al (Liu et al., 2018). utilized a 3D point cloud and spectral clustering to semantically segment Ixora, Brassica, Wheat, and Basic plants. Then, they further segmented down to each of the plant part’s leaves and stem. Their technique was unique, and their framework was capable of segmenting a variety of plant species. Valliammal et al (Valliammal and Geethalakshmi, 2012). proposed a study where they applied wavelet transformation and fuzzy clustering to segment leaves. They was able to provide good segmentation results while achieving high identification of the leaf’s edges. Another study (Wang et al., 2018) proposed a framework that combined the Sobel operator and the Chan-Vese model to segment cucumber leaves with complex background and occlusion issues. A downside to these algorithms is that they are sensitive to high levels of noise and gray inhomogeneity and are difficult to determine the initial parameters (Li et al., 2020).

Researchers have begun using Convolutional Neural Networks (CNNs) in their applications since 2012 due to their promising performance in semantic and instance segmentation (Jiang and Li, 2020) by utilizing the foreground object’s features. Most applications combine CNNs with deep learning libraries such as Caffe (Pound et al., 2017), TensorFlow (Koh et al., 2021), PyTorch (Zhou et al., 2021), and Keras (Gong et al., 2021) for their analysis. Researchers have attempted to utilize neural network-based segmentation algorithms to count plant organs that have replaced some traditional-based and clustering-based algorithms. Most of these neural network-based algorithms (Bolya et al., 2019; Chen et al., 2020; Kirillov et al., 2020) require plenty of images with pixel-level annotation and available training data. Neural network-based algorithms are also used for data augmentation strategies for plant organ identification, segmentation, and counting (Das Choudhury et al., 2017; Das Choudhury et al., 2020; Mazis et al., 2020). Studies that have used CNNs have shown to have achieved accuracies from 87% to 99% for stress-based application and classification (Mohanty et al., 2016; Cruz et al., 2017; DeChant et al., 2017; Fuentes et al., 2017; Lu et al., 2017; Wang et al., 2017; Barbedo, 2018; Barbedo, 2018; Ferentinos, 2018; Liu et al., 2018; Suh et al., 2018; Nazki et al., 2020) but with manual or naïve modifications of the binary masks after processing.

### 2.3 Cosegmentation algorithms

Merdassi et al. (Merdassi et al., 2020), categorized cosegmentation algorithms into eight categories: Markov Random Fields-based Cosegmentation (MRF-Coseg), Co-Saliency-based Cosegmentation (CoS-Coseg), Image Decomposition-based Cosegmentation (ID-Coseg), Random Walker-based Cosegmentation (RW-Coseg), Maps-based Cosegmentation (M-Coseg), Active Contours-based Cosegmentation (AC-Coseg), Clustering-based Cosegmentation (Cl-Coseg), and Deep Learning-based Cosegmentation (DL-Coseg). They quantified that almost all algorithms in these categories used only color and texture features. This presents an issue because if the algorithms are intended to only recognize color and features, then the algorithm cannot detect a heterogeneous object that may consist of multiple distinctive regions. Complex, objects, such as plants, are heterogenous and can vary in color and texture as time progresses.

Several DL-Coseg studies (Hsu et al., 2018; Li et al., 2018; Meng et al., 2019) have found that using a CNN-based framework is optimal for detecting, extracting, and map-generating an object’s features for a set of images. Hsu (Hsu et al., 2019) used CNNs to detect co-peaks for an image pair and its features to determine segmentation masks. Li (Li et al., 2018) utilized a CNN-based Siamese encoder-decoder architecture to extract semantic features of the objects in a set of images. Hsu (Hsu et al., 2018) generated heat maps for each image and transformed the results for cosegmentation via dense CRFs. These algorithms require a large-scale set of images to achieve results, but that is extremely time-consuming. Although some algorithms tackle this problem, they end up being semi-serviced learning-based methods (Kim et al., 2011; Wang and Liu, 2013).

Recent cosegmentation algorithms have tackled important issues such as occlusion (Jerripothula et al., 2021; Meng and Zhao, 2022) by leveraging a combination of techniques to aid in object detection. Also, the literature supports incipient cosegmentation applications in pancreas research (Liu et al., 2022). Other sources of imagery, such as UAV-based high throughput platforms (Rico et al., 2020; Rico et al., 2021) or applications to improve the predictability of phenotypes (Sarzaeim et al., 2022; Sarzaeim et al., 2023), illustrate the potential of the use of cosegmentation algorithms and datasets for a variety of object types.

### 2.4 Cosegmentation datasets

Several datasets to demonstrate the efficacy of cosegmentation algorithms have been proposed in the literature, including iCoseg (Batra et al., 2010), MSRC (Winn et al., 2005), Internet (Rubinstein et al., 2013), Flickr-MFC (Kim and Xing, 2012), and PASCAL-VOC (Everingham et al., 2010). However, these datasets do not reflect the complexity in many application domains where the shape of the objects change over time (temporality), the objects are imaged in different imaging sensors (modality), and under different environmental conditions. These datasets are extremely big in image count that it is difficult to parse or even provide ground truth data for them. Furthermore, most of the objects in the datasets are random and have yet to be used in domain-specific research. The datasets are also limited in their ability to be used in current problems that require temporality, object state change, and multiple modalities for a diverse set of data points. CosegPP (Quiñones et al., 2021), on the other hand, contains many features that could be leveraged to aid current problems to advance cosegmentation research in general and in application-specific work–plant phenotyping.

Traditional and learning-based algorithms are not advanced enough to handle a VSTEM dataset since they currently rely on naïve modifications or are unable to leverage the necessary information for deeper plant analysis. Cosegmentation algorithms tend to overcome the issues with traditional and feature-based algorithms and address complex challenges, such as occlusion, but are only efficient for a specific type of dataset. An overview of cosegmentation datasets suggests that they may not be useful for domain-specific applications and motivates the need for domain-specific datasets. Our work introduces an end-to-end unsupervised deep learning framework and a VSTEM dataset that is 1) high dimensional, and 2) contains a small number of (7 to 14) images. Furthermore, it is the first cosegmentation-based algorithm proposed and tested for plant phenotyping.

## 3 Methods

**Figure 2:**
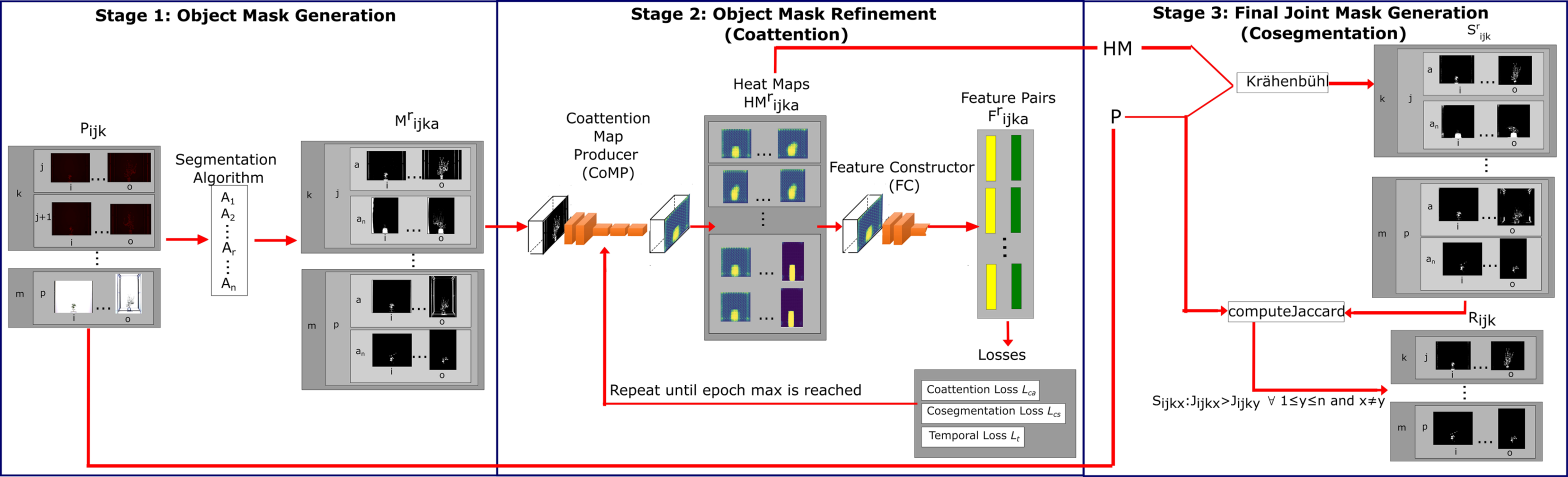
Block diagram of OSC-CO^2^. This method contains three stages. The Object Mask Generation stage takes imagery, P, and produces binary imagery, M. The Object Mask Refinement stage takes the imagery, M, and processes it through the Coattention Map Producer, CoMP, generating an HM set of heat maps. Then, the maps go through the Feature Constructor, FC, to produces a two-column tensor of feature information of the background and object. The two-column tensor is used as input to the coattention loss ( $l_{ca}$ ) (Hsu et al., 2018), and our novel cosegmentation (l_cs_) and temporal ( $l_{t}$ ) loss. This stage stops after the epoch max is reached. For the Final Joint Mask Generation stage, we adopted dense conditional random fields (CRFs) as our refinement taking HM and P as input for Krähenbühl’s algorithm, then giving a final dataset, R, after computing Jaccard index similarity between S and P.

OSC-CO^2^ uses an information fusion approach by leveraging the outputs from multiple segmentation and cosegmentation methods to learn and refine the segmentation of VSTEM images. Specifically, for the images in a VSTEM image dataset, the object of interest will exhibit a variety of state changes over time (but captured at specific temporal points) and is captured in multiple imaging modalities at multiple views. All the available information can be leveraged to aid segmentation. Iteratively, the whole VSTEM dataset is segmented by OSC-CO^2^ by determining the object using coattention and then cosegmenting the object with one pair of images. A novelty in OSC-CO^2^ is that, unlike traditional CNNs, it is completely unsupervised, which suggests that no additional data annotations are needed. OSC-CO^2^ is implemented in three stages: Object Mask Generation (OMG), Object Mask Refinement (OMR), and Final Joint Mask Generation (FJMG). **Figure 2** shows the three stages and how they are processing the dataset input and sending information across stages to generate the metric output.

### 3.1 Problem definition

The problem including VSTEM imagery can be formally defined as follows:

Given a plant P, imaged at m time points, o modalities, and in p side views, i.e., $P=\left\{P_{ijk}\right\},1\leq i\leq m,1\leq j\leq o,1\leq k\leq p$ , where $P_{ijk}\;$ is the image of plant P at time i , view j , and modality k , determine $R=\left\{R_{ijk}\;\right\},1\leq i\leq 0,1\leq j\leq p,1\leq k\leq m$ , where $R_{ijk}$ is the final segmented mask for the plant $P_{ijk}$ .

The proposed OSC-CO^2^ algorithm incorporates a dynamic and expandable approach using coattention and cosegmentation analytics. It consists of three stages: (1) Object Mask Generation, (2) Object Mask Refinement, and (3) Final Joint Mask Generation. This model is designed to handle high-throughput image datasets to generate accurate separation of dynamic and evolving objects using a deep learning framework. This approach generates an ensemble of binary masks for a VSTEM image set and addresses some common challenges in segmentation, including background noise and the evolution of the object’s morphology. The proposed OSC-CO^2^ framework is summarized in **Algorithm 1** below.

**Algorithm 1.** Proposed OSC-CO^2^ framework

**Input:** $P=\left\{P_{ijk}\right\},\;1\leq i\leq o,1\leq j\leq p,1\leq k\leq m$ , where $P_{ijk}$ is the image of plant P at time *I*, in view j , modality k . $C_{jk}$ is the class at modality *k*, and view *j*. $A=\;\{A_{1},A_{2},\ldots ,A_{r,}\ldots ,A_{n}\},\;$ where $A_{r}$ is the $r^{th}$ segmentation algorithm that takes a plant image and generates its mask. **Output:** R={R}, 1≤i≤m,1≤j≤o,1≤k≤p , where $R_{ijka}$ is the refined binary masks for the plant $P_{ijka}$ ** Stage 1: Object Mask Generation** (P, A) ** Begin** **For** 1 ≤a≤n **do** **For** 1 ≤i≤o **do** **For** 1 ≤j≤p **do** **For** 1 ≤k≤m **do** $M_{ijk}^{r}=Segment\left(A_{i},P_{ijk}\right);$ **Return** (M) **End** **Stage 2: Object Mask Refinement** (M) **Begin** **For** 1≤i≤o **do** **For** 1≤j≤p **do** **For** 1≤k≤m **do** **For** 1 ≤a≤n **do** **Begin/**/process the image temporal sequence. Initialize (CoMP, FC) **For** 1≤k≤m−1 **do** **Begin** $I_{prev}=M_{i,j,k}^{r};I_{current}=M_{i,j,\left(k+1\right)}^{r}$ $L_{ca}=L_{co}=L_{t}=0$ **Repeat** $HM_{i,j,ka}^{r}=COMP\left(I_{prev},\;I_{current}\right)$ //heat maps $F_{i,j,ka}^{r}=FC\left(I_{prev},\;I_{current}\right)$ //features Compute $L_{ca}$ using Eq. (1) Compute $L_{cs}$ using Eq. (4) Compute $L_{t}$ using Eq. (7) $Loss_{Total}$ = $1*L_{ca}$ + $0.5*L_{co}+0.5*L_{t}$ Backpropagate loss and update weights **Until epoch max is reached** **End** **End** **End** **Return** (HM) **End** **Stage 3: Final Joint Mask Generation** (P, HM) **For** 1≤i≤o **do** **For** 1≤j≤p **do** **For** 1≤k≤m **do** **For** 1 ≤a≤n **do** $S_{ijk}^{r}\;=\;Krähenbühl\left(P_{ijk},\;HM_{ijk}^{r}\right)$ $J_{ijk}^{r}\;=\;computeJaccard\left(S_{ijk}^{r},\;P_{ijk}\right)$ **End** $R_{ijk}\;=\;S_{ijkx}:J_{ijkx}>J_{ijky}\;\forall \;1\leq y\leq n\;\text{and }x\neq y$ **End** **End** **End** **Return** (*R*)

### 3.2 Overview

Given a set of images of a single plant P in different modalities and views at different time points, we start with a set of basic segmentation algorithms to generate initial masks. Segmented images in the temporal sequence are reconciled in order using deep neural networks with novel loss functions. The final segmentation results are derived by analyzing the refined segmentation results from different algorithms. **Figure 2** shows an overview of the OSC-CO^2^ framework. As shown in the figure, OSC-CO^2^ consists of three key stages: initial mask generation, mask refinement, and final mask creation.

### 3.3 Object mask generation

The goal of the OMG stage is to generate the initial segmentations for all the images of the plant, including all modalities, views, and time points. The masks are generated for each segmentation algorithm and are used in Stage 2 to refine them. In the OMG stage, the input images, P, are processed through a set of algorithms selected by the user defines to produce a set of binary images, M, for all the algorithms. This stage has no limit to the number of algorithms and images that it can handle, but it could be limited by a computer’s processing power.

### 3.4 Object mask refinement

**Figure 3:**
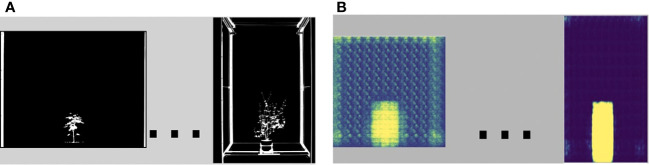
Our heat maps follow the standard color definition where purple and blue is the “coldest” (weak object prediction), and red and yellow is the “hottest” (strong object prediction). **(A)** Shows some binary imagery with its heat maps **(B)**.

The OMR stage takes the binary imagery, M, through a neural network called the Coattention Map Producer, (CoMP), to create heat maps for each image. Our definition of a heat map is shown in **Figure 3**. The heat maps are passed through another network called the Feature Constructor (FC) that computes the features of the estimated objects and the background. The Coattention Map Producer CoMP learns by optimizing multiple loss functions designed to address the challenges in cosegmenting a VSTEM dataset with evolving objects. The three functions are temporal, cosegmentation, and coattention loss. Temporal loss measures the inter-image object difference defined by the distance between the feature pairs of the current image and that of the previously computed image. The cosegmentation loss measures the foreground-background discrepancy within each image. The third is coattention loss adapted from (Hsu et al., 2018), which enhances inter-image object similarity and intra-image figure-ground distinctness per image. Finally, the P and HM imagery are inputs to the FJMG stage to the dense conditional random fields (CRFs) cosegmentation algorithm to produce our framework’s final joint masks, R.

**Figure 4:**
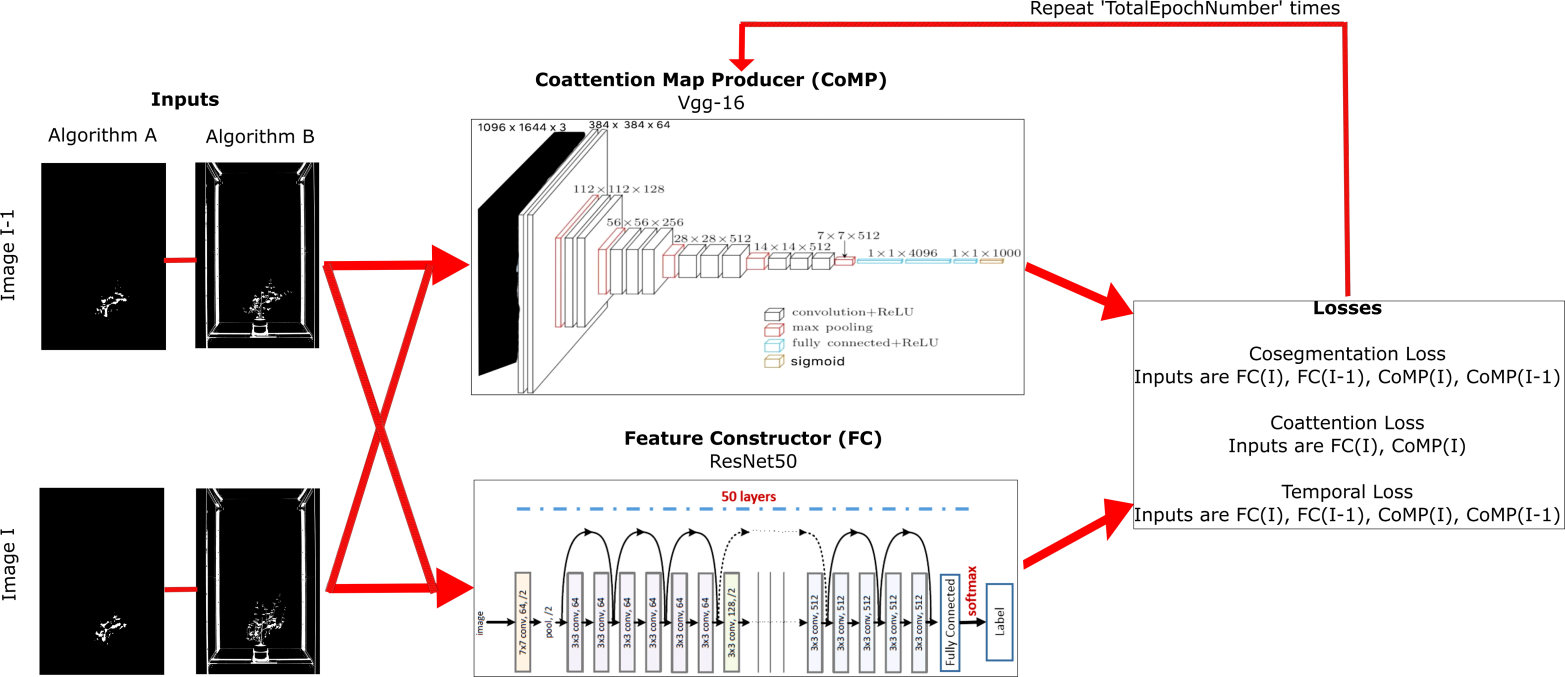
A detailed block diagram of Stage 2: Object Mask Refinement using Coattention. The inputs are simplified in this block diagram to show only the previous and current image with its corresponding imagery for the selected algorithms. These images are inputs to the CoMP and the FC. The generated heat maps from CoMP, and the numerical pairs from FC are used to compute the three losses. These losses are propagated back into CoMP, and this process repeats the number of epochs that is defined in the architecture.

As shown in **Figure 4**, the OMR module is composed of two collaborative CNN modules to produce the heat maps (heat maps that differentiate between the object and background) and feature pairs (descriptive correlation between an image’s foreground and background). They are described below.

• Coattention Map Producer (CoMP): This module produces heat maps.
• Feature Constructor (FC): Generates feature information for the object and background in each image that can be used by the loss functions for optimization.

The details of the modules and their architecture are described in detail below.

#### 3.4.1 Coattention map producer

The CoMP is a fully convolutional network (FCN) (Long, 2015) that has a modified ReLu layer to avoid modifying the data directly and avoid allocating any additional memory. An FCN was used since the architecture does not contain any dense layers, meaning the FCN can handle a wide range of image sizes since all connections are local. This is useful for VSTEM datasets that contain hundreds of images due to the temporal resolution. For each input image to CoMP , it estimates its heat map, i.e., $HM_{ijk}^{r}=CoMP\left(M_{ijk}^{r}\right)$ . We used the VGG-16 (Simonyan and Zisserman, 2015) setting of the FCN (Long et al., 2020) to create CoMP . Following (Hsu et al., 2018) we replaced the last activation function *softmax* layer with a *sigmoid* function layer which provides the heat maps as output. We also kept the learning rate set to $10^{-6}$ and fixed it during the optimization process following (Hsu et al., 2018).

#### 3.4.2 Feature constructor

FC is a Resnet50 (He et al., 2016) that takes in a segmented image, $HM_{ijk}^{r},$ and computes the semantic features object ( $I_{n}^{o}$ ) and background ( $I_{n}^{b}$ ) using Equations 2 and 3. A Resnet50 architecture was used since it has many layers that can be trained easily without increasing the training error while overcoming the vanishing gradient problem in the VGG-16 architecture in CoMP . This approach is useful for our imagery since our evolving objects contain many parts, textures, shadows, and colors. These features are from the last fully connected layer of FC (Hsu et al., 2018) since VGG-16 (Simonyan and Zisserman, 2015) sometimes suffers from the vanishing gradient problem. Our method recognizes that early heat maps are too unstable and compensates with resilient adjustments. Furthermore, FC is an off-the-shelf model pre-trained with ImageNet (Deng et al., 2009). We have set the features extracted in FC as inputs to the last fully connected layer.

#### 3.4.3 Loss functions

A novel contribution of OSC-CO^2^ is a loss function developed to address the unique properties of the VSTEM datasets. The overall loss function is defined as


$$L_{EMVS}=1*L_{ca}+0.5*L_{cs}+0.5*L_{t},$$

where $L_{cs}$ is the coattention loss, $L_{cs}$ is the cosegmentation loss, and $L_{t}$ is the temporal loss.

The coattention loss is designed to enhance both inter-image object similarity and intra-image figure-ground distinctness in each image, aiding in extracting our object type. Our novel cosegmentation loss optimizes the images by using the object and background’s features for a high foreground object similarity across the output masks and a high foreground-background dissimilarity within each image. This loss will aid in extracting information across the different views and modalities. Our novel temporal loss optimizes the similarity of the foreground objects across two sequential images. This loss will help in providing information about a specific object’s type for each environmental condition. All these losses target all the aspects of a VSTEM dataset.

##### 3.4.3.1 Coattention Loss

The coattention loss is defined by Hsu et al. (2018) and is meant to guide CoMP ’s training of optimal coattention masks by referring to the current object and background features that are computed by FC . The loss function is defined below:


(1) $$l_{c}\left({\left\{I_{n}\right\}}_{n=1}^{N};w\right)=-\sum_{i=1}^{N}\sum_{j\neq 1}\text{log}(p_{ij})$$

##### 3.4.3.2 Cosegmentation Loss

One assumption we made about the VSTEM dataset is that the object relatively stays in the same position but grows outwards. We exploit this advantage to aid in object alignment. The proposed cosegmentation loss is designed to guide CoMP to generate a high foreground object similarity across the images and high foreground-background dissimilarity within each image. Given the current and previously computed image pairs $\left(I_{A},\;I_{B}\right)$ with the current and previously computed generated mask pairs from $CoMP\;\left(S_{A},\;S_{B}\right)$ , we produce the object and background features. We generate the object $\left(I_{n}^{o}\right)$ and background $\left(I_{n}^{b}\right)$ features by


(2) $$I_{n}^{o}=FC\left(I_{n}⊗S_{n}\right)\text{ and}$$

(3) $$I_{n}^{b}=FC\left(I_{n}⊗\left(1-S_{n}\right)\right)\;for\;n\in \left\{A,B\right\},$$

where ⊗ denotes the pixel-wise multiplication between the two operands. The cosegmentation loss $\left(L_{cs}\right)$ is defined by


(4) $$L_{cs}\left(I_{A},\;I_{B},\;F\right)=d_{AB}^{+}+d_{AB}^{-},$$

where $d_{AB}^{+}$ and is defined as


(5) $$d_{AB}^{+}=\frac{1}{c}{\left\|F\left(I_{A}^{o}\right)-F\left(I_{B}^{o}\right)\right\|}^{2}\text{ and}$$

(6) $$d_{AB}^{-}=\max\;(0,m-\left(\frac{1}{2c}\left(\left|\left|F\left(I_{A}^{o}\right)-F\left(I_{A}^{b}\right)|{|}^{2}+\right|\right|F\left(I_{B}^{o}\right)-F\left(I_{B}^{b}\right)|{|}^{2}\right)\right)$$

The margin m enlarges the difference between classes to enhance classification ability. If the margin is too large, the probabilities become unreliable, leading to a large loss for almost all samples (Zhang et al., 2019). For our framework, it is set to 2 as the cutoff threshold. Eq. (5) aims to minimize inter-image foreground object distinctiveness, and Eq. (6) maximizes the intra-image foreground-background discrepancy. Even though the cosegmentation loss $L_{cs}$ is like the loss described in (Chen et al., 2020), there is a significant difference. Our cosegmentation loss measures the mean squared error (MSE) (squared L2 norm) between each element in the input x and target y (the variable definition used by PyTorch) instead of using the dimension of the features as the constant c . In addition, since MSE penalizes prediction that is far away from the previously computed by applying a squared operator, we used that as our criterion to stop the computation of our losses when near convergence. To the best of our knowledge, computing a loss with results from previous iterations to model the temporal effect has not been explored.

##### 3.4.3.3 Temporal Loss

This loss assumes that there exists an object that changes in shape due to environmental conditions, thus, insinuating a discrepancy between the foreground and background as time progresses. The temporal loss is designed to measure the inter-image object distance between the current and previously computed image feature pairs of $\left(I_{A},\;I_{B}\right)$ . We generate the object $\left(I_{n}^{o}\right)$ and background $\left(I_{n}^{b}\right)$ features based on Eq. (2) and (3). The temporal loss $\left(L_{t}\right)$ is defined by


(7) $$L_{t}\left({\left\{I_{n}\right\}}_{n=1}^{N}\right)=-\sum_{i=1}^{N}\sum_{j\neq i}\text{log}\left(p_{ij}\right),$$

where $p_{ij}$ is defined as


(8) $$p_{ij}=\frac{\exp\left(-ob_{ij}^{+}\right)}{\exp\left(-ob_{ij}^{+}\right)+\exp\left(-ob_{ij}^{-}\right)},$$

(9) $$ob_{ij}^{+}=\frac{1}{c}{\left|\left|F\left(I_{B,i}^{o}\right)-F\left(I_{B,j}^{o}\right)\right|\right|}^{2}\;,\text{ and}$$

(10) $$ob_{ij}^{-}=\frac{1}{c}(||F{\left(I_{A,i}^{o}-F\left(I_{A,j}^{o}\right)||\right)}^{2}$$

CoMP generates heat maps to optimize low inter-image object distances for both current and previously computed feature pairs using Eq. (9) and (10). Our temporal loss is motivated by Hsu’s (Hsu et al., 2018) coattention loss, but the difference is that ours ignores the intra-image figure-ground dissimilarity and computes the inter-image object distance with the current and previously computed image feature pairs.

### 3.5 Final joint mask generation

This stage uses the dense CRF approach proposed in (Krähenbühl and Koltun, 2011), where the unary and the pairwise terms are set to the two heat maps generated from the results of two segmentation algorithms and bilateral filtering, respectively. For each pixel in the heat maps, we define a probability that the pixel belongs to undefined classes. The hyperparameters for the network are summarized in **Table 1**. This stage outputs the final binary masks, R, by computing and selecting the mask with the highest Jaccard index similarity between the plant imagery dataset, P, and the segmented masks dataset, (S), using the dense CRF approach (Krähenbühl and Koltun, 2011).

**Table 1:** Hyperparameter values of the dense CR network approach (Gong et al., 2021).

Hyperparameter	Value
Weight of the bilateral term	10
Spatial standard deviation	80
RGB standard deviation	13
Weight of the spatial term	3.0
Spatial standard deviation	3
Number of iterations	5.0

### 3.6 Optimization process

OSC-CO^2^ uses the ADAM optimizer to derive its hyperparameters due to its widespread use and its rapid convergence (Hsu et al., 2018; Mehta et al., 2019; Melinte and Vladareanu, 2020) properties. The final parameters determined by ADAM include a 0.01 learning rate and a 0.0005 weight decay for CoMP' s parameters. At the start of processing each one-pair of images, the optimizer sets all the gradients to zero.

### 3.7 The data repository creation

The VSTEM imagery used to evaluate the performance of OSC-CO^2^ is based on the CosegPP data repository (Quiñones et al., 2021). The data repository has plant images with a large inter-class variation and background noise. The images were captured using the LemnaTec Scanalyzer at the University of Nebraska-Lincoln, USA. It contains two buckwheat plants, where one underwent drought stress, and the other remained the control, and two sunflower plants, where one underwent drought stress, and the other remained the control. Each plant represents a dataset that has four side views (0°, 72°, 144°, 216°), and three modalities (fluorescence, infrared, and visible) with 7 to 14 time points.

We created an extension of CosegPP’s datasets, which we will refer to as CosegPP+, by processing all four plant datasets through segmentation using Otsu’s method (Otsu, 1979) and cosegmentation using Subdiscover (Meng et al., 2016). These two methods were chosen since (Quiñones et al., 2021) defined these as the top methods for being able to segment some of the challenging features of computer vision. CosegPP+ is publicly available at https://doi.org/10.5281/zenodo.6863013.

We replaced the original images with the outputs generated by Otsu’s method and Subdiscover. Meaning that each time point i will have at most a binary images where a is the number of algorithms (i.e., Otsu’s method and Subdiscover) used. Some groups do not contain Subdiscover binary masks due to the method’s limitation in not being able to segment the original images.

### 3.8 Implementation

OSC-CO^2^ allows for the dynamic input of epoch runs, but we used 10 epoch runs for CosegPP+. It is worth noting that CosegPP began overfitting after 7 epochs. OSC-CO^2^ also requires a minimum 2 epochs to allow for the heat maps to generate stable proposals and generate the coattention loss based on pixel-wise averaging of the masks. The cosegmentation loss and temporal loss activates at the final epoch. The batch size is set to a (the number of algorithms used as input with their binary masks). Also, all input images are resized to 384 × 384 pixel resolution prior to subsequent processing because FC can only be applied to images of the same size while using 3×3 kernels with a stride of 3 and 3 for height and width, respectively, and with an initial learning rate of 0.001. After the cosegmentation, we resized the images back to their original sizes for performance evaluation.

## 4 Results and discussion

In this section, we describe the dataset, performance metrics, experimental design, and evaluation results for OSC-CO^2^. The results are compared with several existing methods to demonstrate the efficacy of OSC-CO^2^.

### 4.1 Evaluation metrics

Our experiment uses two widely used metrics, Precision (P) and Jaccard index’s (J) similarity (also known as IoU) to evaluate the final estimated masks for each time point. Precision is the measurement that identifies the percentage of correctly segmented pixels. Jaccard index is the measurement for the intersection area ratio between the detected object and ground truth. Both metrics will range from 0 to 1 where 1 is the ideal value. We chose these two metrics due to their continuous use in coattention and cosegmentation analytics (Dong et al., 2015; Meng et al., 2016; Wang and Shen, 2016; Li et al., 2018; Merdassi et al., 2020).

### 4.2 Quantitative results

**Table 2** summarizes the performance of OSC-CO^2^ on the CosegPP+ data repository. The precision and Jaccard index similarity scores for the four plants are presented for each of the three modalities (visible, fluorescence, and infrared). For each modality, the scores for each of the four views are also reported. Finally, the average scores for each plant over all the views and for all the modalities are presented. OSC-CO^2^ produces the highest precision and Jaccard index similarity scores for the fluorescence and visible modality for the buckwheat species and high scores for the infrared modality for the sunflower species.

**Table 2:** The performance of our OSC-CO^2^ on the CosegPP+ data repository.

		Fluo 0°	Fluo 72°	Fluo 144°	Fluo 216°	IR 0°	IR 72°	IR 144°	IR 216°	Vis 0°	Vis 72°	Vis 144°	Vis 216°	Fluo Avg	IR Avg	Vis Avg	All Avg
*BC*	**P**	0.93	0.93	0.93	0.92	0.90	0.90	0.90	0.90	0.99	0.99	0.99	0.99	**0.93**	0.90	**0.99**	**0.94**
	**J**	0.39	0.39	0.39	0.38	0.32	0.31	0.29	0.29	0.37	0.42	0.35	0.33	**0.39**	0.31	0.37	**0.36**
*BD*	**P**	0.92	0.92	0.93	0.93	0.89	0.89	0.89	0.89	0.98	0.95	0.98	0.97	**0.93**	0.89	**0.97**	**0.93**
	**J**	0.43	0.44	0.47	0.46	0.35	0.35	0.34	0.34	0.33	0.27	0.30	0.38	**0.45**	0.35	0.32	**0.37**
*SC*	**P**	0.93	0.86	0.86	0.87	0.92	0.95	0.95	0.95	0.91	0.90	0.92	0.91	**0.88**	**0.95**	0.91	**0.91**
	**J**	0.36	0.31	0.32	0.34	0.41	0.37	0.37	0.36	0.43	0.34	0.44	0.43	**0.33**	0.38	**0.41**	**0.37**
*SD*	**P**	0.95	0.88	0.88	0.91	0.98	0.98	0.99	0.98	0.89	0.92	0.92	0.92	**0.91**	**0.99**	0.92	**0.94**
	**J**	0.23	0.27	0.28	0.27	0.34	0.31	0.31	0.29	0.27	0.26	0.26	0.35	**0.27**	**0.32**	0.29	**0.29**

BC, Buckwheat (Control); BD, Buckwheat (Drought); SC, Sunflower (Control); SD, Sunflower (Drought); P, Precision; J, Jaccard index similarity; Vis, Visible imagery; Fluo, Fluorescence imagery; IR, Infrared imagery. The best average Precision and Jaccard index similarity per dataset are shown in bold.

**Table 3** compares the performance of OSC-CO^2^ against Otsu’s method, a widely used algorithm in the plant phenotyping domain, and Subdiscover, a leading cosegmentation method. The performance results for Otsu’s algorithm and Subdiscover are derived from our previous research (Quiñones et al., 2021). **Table 3A** summarizes the effectiveness of our method, OSC-CO^2^, under normal growth conditions and under drought for buckwheat images. **Table 3A** shows that the performance of OSC-CO^2^ is comparable to other algorithms based on the precision score; it is slightly lower in fluorescence and infrared modality but the same or higher for visible imagery. However, the Jaccard index similarity measures for OSC-CO^2^ are significantly superior to other algorithms. This implies that OSC-CO^2^ can properly detect the object’s pixels, but at the expense of a very slight reduction in precision.

**Table 3:** The comparative evaluation of OSC-CO^2^ with Otsu’s algorithm and Subdiscover.

A. Performance comparison on buckwheat species												
	*Buckwheat (Control)*						*Buckwheat (Drought)*					
	Fluo		IR		Vis		Fluo		IR		Vis	
	P	J	P	J	P	J	P	J	P	J	P	J
**Otsu**	**0.97**	0.38	0.90	0.05	0.93	0.14	**0.97**	0.37	**0.90**	0.05	0.93	0.14
**Subdiscover**	–	–	–	–	**0.99**	0.34	–	–	–	–	**0.97**	0.23
**OSC-CO^2^ (ours)**	0.93	**0.39**	**0.91**	**0.31**	**0.99**	**0.37**	0.93	**0.45**	0.89	**0.35**	**0.97**	**0.32**

B. Performance comparison on sunflower species.												
	*Sunflower (Control)*						*Sunflower (Drought)*					
	Fluo		IR		Vis		Fluo		IR		Vis	
	P	J	P	J	P	J	P	J	P	J	P	J
**Otsu**	0.87	0.26	0.91	0.31	0.92	0.30	0.87	0.12	0.93	0.20	0.93	0.14
**Subdiscover**	0.84	0.10	**0.94**	0.19	**0.98**	**0.47**	0.84	0.00	**0.98**	0.09	**0.98**	0.16
**OSC-CO^2^ (ours)**	**0.92**	**0.39**	0.92	**0.35**	0.96	0.37	**0.92**	**0.36**	0.94	**0.34**	0.95	**0.35**

BC, Buckwheat (Control); BD, Buckwheat (Drought); SC, Sunflower (Control); SD, Sunflower (Drought); P, Precision; J, Jaccard index similarity; Vis, Visible imagery; Fluo. Fluorescence imagery; IR. Infrared imagery. The best average Precision and Jaccard index similarity per dataset are shown in bold. “—” means the algorithm did not generate an output.

**Table 3B** compares the performance of OSC-CO^2^ for the sunflower species. The results are like those for the buckwheat species. The performance for the fluorescence and infrared modality is slightly lower for precision, but significantly better overall for Jaccard index similarity. However, Subdiscover clearly outperformed OSC-CO^2^ in the visible modality. This may be due to the inconsistent appearance of sunflower images due to the presence of flowers (yellow) with green stems and leaves.

### 4.3 Qualitative results

**Figure 5:**
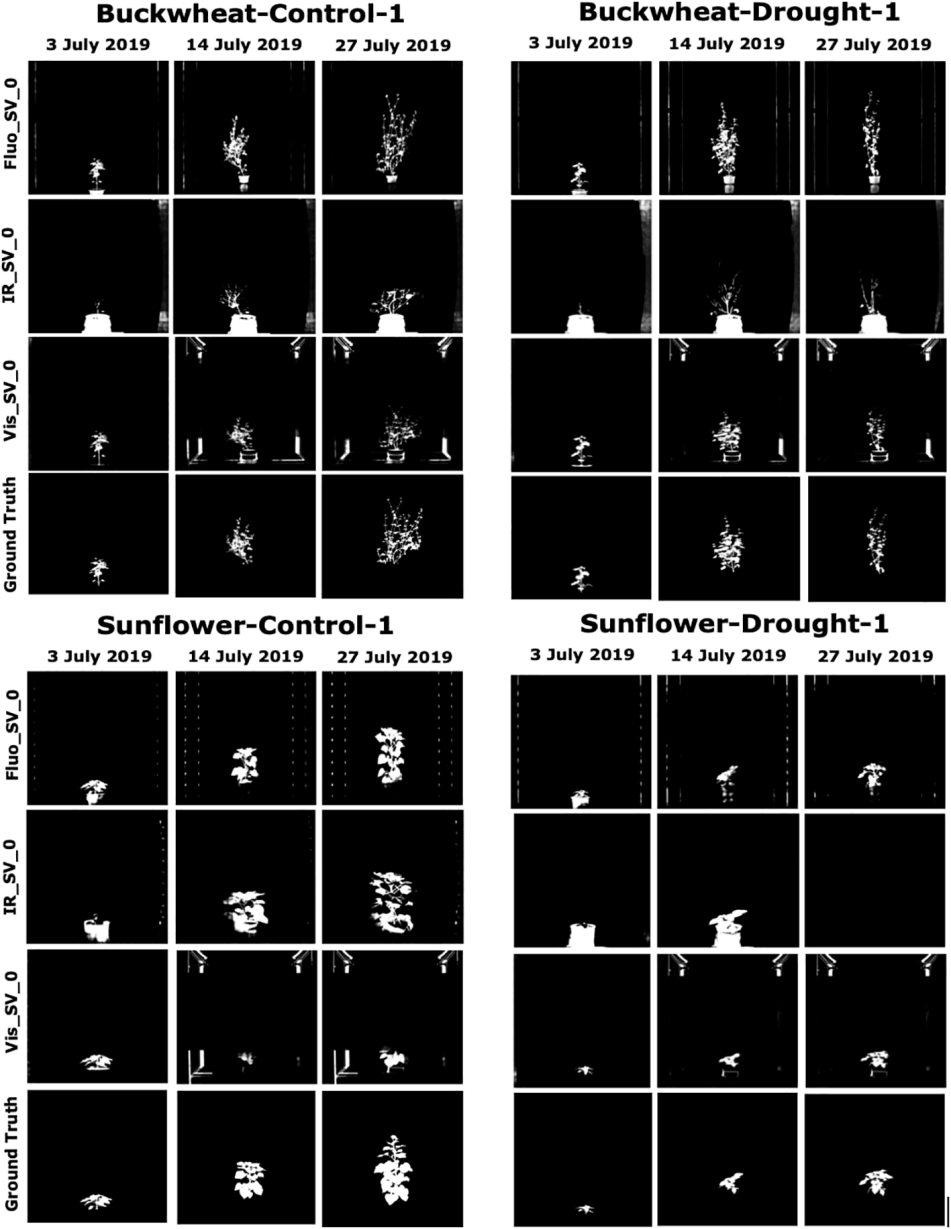
Illustration of qualitative performance of OSC-CO^2^ on the CosegPP data repository. This preview shows only three temporal points (start, middle, end), and one side view for all modalities.

**Figure 5** shows some sample images from the CosegPP+ data repository and their corresponding segmentations generated by OSC-CO^2^. For the buckwheat images, OSC-CO^2^ removes most of the background imaging chamber (background noise in plant phenotyping) while leaving the object (plant) intact. However, the infrared modality was not as accurate in this case. Sunflower images show similar patterns. However, it is noticeable with the Visible light modality that it came at a cost by removing most of the plant itself from the object since those temporal points began to include the flowers. Furthermore, the sunflower displays an empty result for the drought environmental conditions on the last temporal point leading to the assumption that it overfitted too soon. Thus, suggesting the future work of a dynamic epoch cutoff.

As evident from **Figure 5**, the sunflower plants tend to have thicker stems and hence, generate more discernable infrared imagery, resulting in better segmentation accuracy than buckwheat. Furthermore, since buckwheat plants were imaged during the vegetative state only, they were green throughout the imaging period. In contrast, sunflower plants have yellow flowers, sometimes several, during the later stages of growth. Therefore, visible images for buckwheat were more consistent, leading to the highest accuracy. Similarly, the green organs in buckwheat in fluorescence imagery, which serves as a proxy for chlorophyll level, have higher segmentation accuracy in this modality.

## 5 Conclusion and future work

In this paper, we achieve our first contribution of designing an unsupervised method for cosegmenting binary plant imagery by using CNNs that outperformed previous works (Otsu, 1979; Meng et al., 2016) by improving segmentation accuracy by 3% to 45%. The model has three stages. The first stage is the *Object Mask Generation* which produces the necessary binary imagery from a set of user-defined algorithms. The second stage is the *Object Mask Refinement* which uses FCN32, VGGNet, and ResNet50. We also achieved our second contribution by designing two novel unsupervised cosegmentation and temporal loss for stage two with one unsupervised coattention loss from literature. The third stage is *Final Joint Mask Generation* which refines the binary image output by using the heat maps. The experimental results demonstrate a promising new technique that can learn and enhance binary masks, without training data, to refine the masks leading to higher segmentation accuracy for further object analysis.

Using CNNs for evolving objects at different temporal stages shows promising development in increasing accuracy that it may replace some traditional methods for plant phenotyping. This paper creates an unsupervised coattention and cosegmentation method for high-throughput datasets with defined quantitative and qualitative features that leverage the information from multiple algorithms’ binary output. Within this framework, we have proposed two novel loss functions: cosegmentation and temporal loss that aids the coattention loss by helping the discovery of the foreground object while removing background noise.

For our third contribution, experimental evaluations of OSC-CO^2^ on CosegPP+ demonstrate the method’s great capabilities of being able to recognize the evolving, moving object. This also introduces a base analysis for different types of modalities that are being used more in plant phenotyping analytics. Our method was able to leverage these object features to produce and demonstrate its optimal performance among different modalities and environmental conditions.

This paper is a critical contribution to image segmentation to high-throughput multi-modal image segmentation because it eliminates the need for researchers to perform naïve image pre-processing, such as image cropping that may skew an algorithm’s performance by eliminating the complex aspect of an image that may challenge, and push future algorithmic development.

Future work includes implementing a dynamic epoch cutoff algorithm tailored to dataset varieties in terms of environmental conditions and species. An adjustment of the coattention framework can be made to include the selection of flowers and merge it with the object. This could significantly improve segmentation accuracy. Finally, hyperparameter weights can be implemented for the different dimensions in the dataset so that the algorithm can leverage more of the appropriate dimension for higher segmentation accuracy.

## Data availability statement

The original contributions presented in the study are included in the article/supplementary material, further inquiries can be directed to the corresponding author/s.

## Author contributions

RQ contributed as the first author in constructing and analysing the framework, led the experimental analysis, and writing the manuscript. AS, FMA and SDC contributed in supervising, writing and reviewing the original draft. All authors contributed to the article and approved the submitted version.
